# Dosimetric characterization of whole brain radiotherapy of pediatric patients using modulated proton beams

**DOI:** 10.1120/jacmp.v12i2.3308

**Published:** 2011-01-19

**Authors:** Hosang Jin, Wen Hsi, Daniel Yeung, Zuofeng Li, Nancy P. Mendenhall, Robert B. Marcus

**Affiliations:** ^1^ University of Florida Proton Therapy Institute Jacksonville Florida 32206 USA; ^2^ Procure Treatment Centers Inc. Bloomington Indiana 47404 USA

**Keywords:** proton whole brain radiotherapy, plan evaluation, usage of compensator, dose volume histogram, homogeneity index

## Abstract

This study was designed to investigate dosimetric variations between proton plans with (PPW) and without (PPWO), a compensator for whole brain radiotherapy (WBRT). The retrospective study on PPW and PPWO in Eclipse and XiO systems and photon plans (XP) using controlled segments in Pinnacle system was performed on nine pediatric patients for craniospinal irradiations. DVHs and derived metrics, such as the homogeneity index (HI), the doses to $2\%(D_{2\%})$ and $5\%(D_{5\%})$ volumes, and mean dose $\left(D_{\text{mean}}\right)$ of the whole brain (i.e., PTV), and the organs at risk (OARs) such as lens and skull, were obtained. The PPW plans from both Eclipse and XiO systems uncovered the following advantages: (1) encompassing a cribriform plate area with the 100% isodose line was better than either PPWO or XP, according to calculated two‐dimensional distributions of one patient; (2) the mean value of $D_{5\%}$ for lens was reduced to 23.6% of $D_{P}$ from 54.1% for PPWO or 41.6% for XP; and (3) the mean value of $D_{\text{mean}}$ for skull was reduced to 94.8% of $D_{P}$ from either 98.4% for PPWO or 98.3% for XP. However, the PPW plans also exposed several disadvantages including: (1) the HI of PTV increased to 7.7 from 4.7 for PPWO or 3.7 for XP; (2) $D_{2\%}$ to PTV increased to 108.8% of $D_{P}$ from 104.8% for PPWO or 105.1% for XP; and (3) $D_{5\%}$ to the skull increased to 104.9% of $D_{P}$ from 101.6% for PPWO or 103.4% of for XP. One‐half of the observed variations were caused by different penumbra on lateral profiles and distal fall‐off depth doses of protons in Eclipse and XiO. Because the utilization on the sharp proton distal fall‐off was limited for WBRT, the difference between PPW and PPWO or XP indicated no distinguishable improvement by using a compensator in proton plans.

PACS number: 87.55.‐x

## I. INTRODUCTION

The goal of whole brain radiotherapy (WBRT) using protons as part of craniospinal irradiation (CSI) is to reduce the neuron morbidity while simultaneously maintaining or improving long‐term survival rates.^(1)^ The morbidity and substantial side effects of WBRT are strongly related to the total delivered doses, its uniformity to whole cranium and maximum doses to 2%–5% volume of organs at risk (OARs).^(2)^ With the advantage to stop protons at a defined depth, the CSI treatment using proton beam shows a significant reduction of doses to normal tissues surrounding the spinal cord.

Before evaluating dosimetric variations for proton WBRT, the dosimetric issues of WBRT using a fundamental technique of conventional photon radiotherapy with two opposed lateral fields had to be reviewed. The issues for photon WBRT include:

1) underdosage at the cribriform plate and fossa caused by tighter margin anterior to brain by collimator or reshaped block to reduce doses to lens,^(^ ^2^ ^–^ ^4^ ^)^
2) hair loss (consequently alopecia) resulting from a 10%–15% higher dose than prescription delivered to the scalp due to the flush dose of MV photon passing less thickness of medium,^(2)^ and
3) radiation‐induced cataract and lens opacities due to damaged epithelium on the lens by low‐dose radiation (at level of 20.0 Gy).^(^ ^5^ ^,^ ^6^ ^)^

Doses to cribriform plate and fossa could be improved by using oblique‐lateral proton beams with a compensator to stop protons before the eyes without decreasing the margin of aperture around eyes to reduce doses to the lens.^(5)^ However, the dose nonuniformity of whole cranium using protons with a compensator was found to be similar to the WBRT using two opposite‐lateral photon fields. In addition, doses delivered to whole brain at near distal edge of proton beam and to OARs could vary largely from calculated doses of treatment planning when effects of uncertainties due to distal edge of delivered protons and patient positioning are considered. A further study is needed to investigate those effects.

Therefore, treatment planning of proton WBRT without a compensator was implemented to treat patients in our Institute (University of Florida Proton Therapy Institute (UFPTI)), to improve the dose uniformity of whole brain. However, proton WBRT without a compensator comes with a cost of larger doses to the lens. Additionally, once dose uniformity of whole brain is addressed, the doses to the scalp need to be studied for hair loss. Where the skin on the scalp is less than 1 cm thick, doses to the scalp cannot be accurately calculated by dose calculation algorithms used in treatment planning systems (TPSs) for proton and photon beams;^(^ ^7^ ^–^ ^9^ ^)^ because of that, doses to the skull were calculated as a surrogate for doses to scalp in this study.

Although several research groups demonstrated dosimetric and potential superiority of proton beam therapy to conventional photon beam therapy or intensity‐modulated radiation therapy (IMRT) in CSI cases,^(^ ^2^ ^,^ ^10^ ^–^ ^12^ ^)^ none of those studies directly addressed the usage and effect of a compensator in proton WBRT. In this study, the dosimetric characteristics of proton WBRT were investigated by quantitatively evaluating the dosimetric variations in whole brain and OARs such as lens and skull. The proton plans with and without a compensator were calculated using Eclipse (Varian Medical Systems, Palo Alto, CA) and XiO (Elekta., Stockholm, Sweden) TPSs, while plans of photon were made with improved dose distributions by using controlled multileaf collimator (MLC) segments in Pinnacle^3^ (Philips Medical Systems, Andover, MA) TPS. The same structures were used (through DICOM export) in all of the proton and photon treatment plans for each patient with the same prescription dose to planning target volume (PTV). Dose uniformity by homogeneity index (HI) and dose‐volume‐histogram (DVH) of whole brain, and doses to lens and skull were obtained from the proton WBRT plans with and without a compensator, and further compared with those of plans using photon beams.

## II. MATERIALS AND METHODS

To limit the variations of brain size, this study focuses only on pediatric patients (see Table 1 for PTV volumes). Total doses to whole brain are more restrictive and have bigger impact on the outcome of pediatric CSI. The protocol used for proton WBRT as part of CSI treatment in UFPTI is described first. To quantitatively investigate the dosimetric characteristics of the proton WBRT, a retrospective study using recalculated plans of proton and photon WBRT was performed. Protons were delivered using a double‐scattering technique on an IBA (Ion Beam Applications, Louvain‐la‐Neuve, Belgium) gantry beam line in UFPTI for actual treatments. Recalculated proton plans in the Eclipse TPS used the commission data of this IBA gantry beam, but recalculated proton plans in the XiO TPS used the commission data of a gantry beam line at Midwest Proton Radiotherapy Institute, Bloomington, IN (MPRI). MPRI uses an active uniform‐scanning technique in the gantry beam line. Two different beam lines and TPSs are used to investigate consistency of results. Differences between UFPTI and MPRI proton beam lines are discussed. Finally, metrics such as HI, DVH and doses to 2% or 5% volume used to evaluate the dosimetric characteristics of proton WBRT are described.

**Table 1:** HI and $D_{2\%}$ of the PTV of nine pediatric cranio‐spinal irradiation patients.

		*Eclipse*				*XiO*					
*Patient No.*	*PTV Volume (cc)*	*Without Compensator*		*With Compensator*		*Without Compensator*		*With Compensator*		${\text{Pinnacle}}^{3}$	
		*HI*	$D_{2\%}$	*HI*	$D_{2\%}$	*HI*	$D_{2\%}$	*HI*	$D_{2\%}$	*HI*	$D_{2\%}$
1	1165.4	4.9	24.6	7.7	25.5	3.7	24.3	5.7	24.9	3.1	24.5
2	1250.9	4.7	24.6	6.3	25.1	3.1	24.2	6.6	25.2	3.2	24.5
3	1150.7	5.5	24.8	7.1	25.4	4.0	24.4	5.7	24.9	3.4	24.4
4	1408.2	7.1	25.1	10.1	26.1	4.1	24.4	6.5	25.1	4.1	24.6
5	1234.9	4.5	24.5	9.7	26.2	3.5	24.3	6.3	25.0	3.3	24.6
6	1443.6	7.1	25.1	9.0	25.8	5.0	24.6	7.2	25.2	4.1	24.7
7	1176.9	5.2	24.6	7.6	25.5	3.7	24.3	6.1	25.0	3.7	24.5
8	1288.6	4.7	24.6	9.7	25.9	4.6	24.5	7.0	25.2	3.0	24.5
9	1244.6	4.0	24.4	8.1	25.6	3.9	24.4	11.0	26.3	5.1	24.9
Mean $(D_{2\%}:\%$ to $D_{p}$)^a^	5.3	105.5	8.4	109.8	4.0	104.1	6.9	107.7	3.7	105.1
SD $(D_{2\%}:\%$ to $D_{p}$)^a^	1.1	1.1	1.3	1.5	0.6	0.6	1.6	1.8	0.7	0.6

a Mean values and standard deviations (SDs) of *HI* and $D_{2\%}$ in percentage of $D_{P}$.

### A. Protocol of proton WBRT as part of CSI treatment at UFPTI

Nine pediatric patients who underwent CSI treatments on our institutional review board‐approved proton protocol were selected for this study. The patients were treated with modulated proton beams. They were scanned and treated in a prone position. Because the maximum number of slices is limited to 300, dose calculation in the Eclipse (version 8.2) was done using 2 to 3 mm slice thickness. Prescription dose to a cranial target ranged from 23.4 to 36.0 Gy (relative biological effectiveness (RBE)) with a fractionated dose of 1.8 Gy (RBE), and was renormalized to 23.4 Gy (RBE) for all patients. The RBE‐weighted dose unit of ‘Gy (RBE)’ recommended by the International Commission on Radiation Units and Measurements (ICRU)^(13)^ is equivalent to photon dose measured in Gy, which is obtained by multiplying physical proton dose with RBE. The generally accepted value of 1.1 for the RBE of modulated therapeutic proton beam was used in our Institute, stated as 1.0 Gy of physical proton dose equals to 1.1 Gy (RBE).^(14)^

Currently in our clinic, beams of WBRT targets are designed directly for the CTV with large aperture margin (10.0 mm) without reference to a PTV. Thus, for prescribing and reporting purposes, the CTV was clinically used for the PTV. The rationale of this approach is that the WBRT has small setup uncertainty (~ 3.0 mm) and the 10.0 mm aperture margin is large enough to compensate the uncertainty. For the adjustment of depth margins for proton beam therapy, the distal margin was calculated with 1.5% of proton range to account for Hounsfield unit (HU)‐stopping power conversion uncertainty^(15)^ by considering relatively small range perturbation of proton beam in WBRT. Then, 2.0 mm of distal margin was added to the proton beam range to assure the dose coverage of PTV and doses to OARs located at distal end of proton beams were examined. Relatively large proximal margin of 10.0 mm was used due to full modulation of proton beams to the skin. With those margins, the dose distributions and DVHs were calculated in both Eclipse and XiO TPSs.

The prescription of proton plans in Eclipse was made such that the 95% of PTV received the prescribed dose $\left(V_{D=23.4\;\text{Gy}(\text{RBE})}(\text{PTV})\geq 95\%\right)$. It should be noted that the volumetric prescription was not available for proton plans in XiO and photon plans in Pinnacle^3^. In XiO, the dose to a reference point (usual at beam isocenter) was initially set to be equivalent to the prescribed dose and renormalized such that 95% of PTV received the prescription dose in DVH. In Pinnacle^3^, at least 95% of PTV volume received the prescribed dose. The Eclipse treatment plans were evaluated by patient‐specific quality assurance (QA) measurements in water, which agreed well with the plans.

For simplicity, the dosimetry on boost dose delivered to the posterior fossa was not discussed in this study. A 25 cm diameter snout was used to minimize the number of proton fields for treating a whole spinal cord. By including second or third cervical vertebra into cranial fields, it allowed 3 cm movement for three junction‐feathering of 1 cm displacement at each feathering. Different sets of compensators for spine fields were used for the junction shifts. A compensator was designed with trapezoid‐shaped pseudo contours within vertebrae, which encompassed the spinal cord for spinal fields, because the pseudo contours eliminated many sharp edges. Smearing of 6.0 mm was used for forming the compensator by considering detected patient motion of up to 5.0 mm and 5.0 mm diameter of a milling tool of compensator.

Proton beams with 15° gantry tilt toward posterior direction provided better dose sparing to the lens.^(5)^ For plan evaluation of each treated patient, DVHs of structural contours (cranium, retina, lens and skull) were generated for cranial fields, while those of spinal cord and vertebra were constructed for spinal fields. In this study, two OARs (lens and skull) were selected and evaluated and the retina was excluded from the analysis since the tolerance dose (~ 45 Gy) is higher than the prescription dose. Planning organ at risk volumes (PRVs) for the OARs were not constructed, because these structures were close to air or surface buildup regions where the dose calculation of TPS has large uncertainty.^(^ ^7^ ^–^ ^9^ ^)^ Note that the PRV concept is thus not used in our Institute for WBRT.

### B. Design of the retrospective study

The retrospective study for recalculating plans of WBRT for modulated proton beams with and without a compensator on the patients was performed in the Eclipse and XiO TPSs, while recalculating plans of WBRT for photons were performed in the Pinnacle^3^ TPS. Contours of structures and CT images used for actual treatment plans in Eclipse were transferred through DICOM into XiO and Pinnacle^3^. For proton plans, two opposite oblique lateral fields were used to improve the lens dose sparing. For photon plans, two opposite lateral fields were used. To improve dose homogeneity within the target, three to four segments were manually assigned to each photon beam using forward planning. All of the treatment plans using proton beams were normalized such that 95% of PTV received the prescribed dose. In some segmented forward photon plans, normalization aggravated the coverage of the PTV and clinically important regions such as the subfrontal regions, and thus they were not normalized. Dose calculations were performed in a calculation grid size of 2.0×2.0×2.0 mm^3^ for all plans.

**Figure 1:**
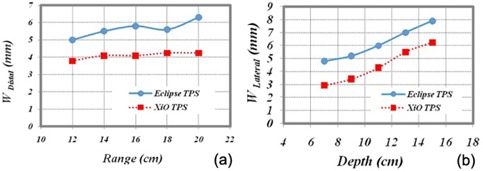
Distal fall‐off and penumbra widths of the Eclipse TPS (UFPTI) and the XiO TPS (MPRI) as a function of (a) range and (b) depth. Shown at left panel are distal fall‐offs (80%–20%) calculated by Eclipse and XiO TPS for protons with modulation width of 5.0 cm and beam ranges between 12.0 cm and 20.0 cm. Shown at right panel are lateral penumbrae calculated by Eclipse and XiO TPS at various depths (7.0, 9.0, 11.0, 13.0 and 15.0 cm) for protons with a beam range of 16.0 cm and a modulation of 10.0 cm. A 10.0 cm aperture margin with 5.0 cm air gap and isocenter at 11.0 cm depth was used for calculations of both distal fall‐off and lateral penumbra.

Details of UFPTI proton gantry beam line used in Eclipse can be found in the publication by Lu and Kooy.^(16)^ Width of distal fall‐off between dose levels of 20%–80% for beam ranges between 12.0 cm and 20.0 cm with protons of 5.0 cm modulation was calculated with a water phantom in the Eclipse TPS (Fig. 1(a). Penumbra widths of lateral profiles at depths from 7.0 cm to 15.0 cm with protons of 16.0 cm range and 10.0 cm modulation were calculated with the water phantom in the Eclipse TPS (Fig. 1(b). A 10.0 cm aperture margin with 5.0 cm air gap and isocenter at 11.0 cm depth was used for all calculations of distal fall‐off and lateral penumbra.

Details about the MPRI gantry beam line can be found in the publication by Farr et al.^(17)^ The active scanning technique used in MPRI rarely requires additional material placed in the proton beam line to laterally spread protons. As a result, the full beam range provided by an accelerator can be used for patient treatments. The calculations of distal fall‐off and lateral penumbra were made in the XiO TPS and plotted in Fig. 1. Sharper distal fall‐off and narrower lateral profiles are seen for the MPRI proton beam line.

### C. Metrics used to evaluate the dosimetric characteristics of WBRT

To perform quantitative comparison of recalculated dose distributions among plans, HI of the PTV was used as one of metrics for the plan comparison.^(18)^ In this study, HI is defined as:

(1) $$HI=\frac{D_{5\%}-D_{95\%}}{D_{p}}\times 100\%,$$

where $D_{5\%},D_{95\%}$, and $D_{P}$ represent doses to 5% and 95% of the PTV and the prescription dose, respectively. Smaller HI represents better homogeneity. $D_{2\%}$ (dose to 2% volume) and $D_{\text{mean}}$ (mean dose) of the PTV were also obtained for comparison.

According to Emami et al.,^(19)^ tolerance of eye lens (TD 5/5 volume; 5% severe complication within five years of radiation completion) for photon dose calculation is 10.0 Gy for whole volume. In CSI cases, the tolerance may be easily exceeded due to field arrangement of two opposed lateral photon beams; therefore, dose to the lens was selected as one of the comparison criteria. $D_{5\%}$ (dose to 5% volume) and $D_{\text{mean}}$ of the lens were accessed. During the treatment planning, the entire or partial lenses are blocked by apertures or collimators to reduce the dose to the lens. This results in possible underdosage to the subfrontal regions at the cribriform plate that potentially causes subfrontal relapse.^(3)^ Therefore, the dose at the subfrontal regions was also investigated and compared by taking dose profiles.

Radiation dose to scalp is of importance in CSI cases due to hair loss. Since inaccuracy in dose calculation of TPSs is widely known in surface regions and it was cumbersome to accurately contour the scalp on the CT images, doses to skull $(D_{5\%}$ and $D_{\text{mean}}$) were investigated in place of scalp doses.

Student's t‐test was performed for analysis of the different treatment techniques with MATLAB statistical toolbox, version 6.0 (The MathWorks, Inc., Natick, MA). A two‐tailed p‐value of < 0.05 was considered statistically significant.

## III. RESULTS & DISCUSSION

### A. Dose distributions of proton and photon plans

Based on simple treatment planning exercises, proton beams without a compensator showed better dose uniformity to whole brain than those with a compensator for cranial fields. Therefore, two oblique lateral proton beams without a compensator were used for WBRT as part of CSI in our Institute.

**Figure 2:**
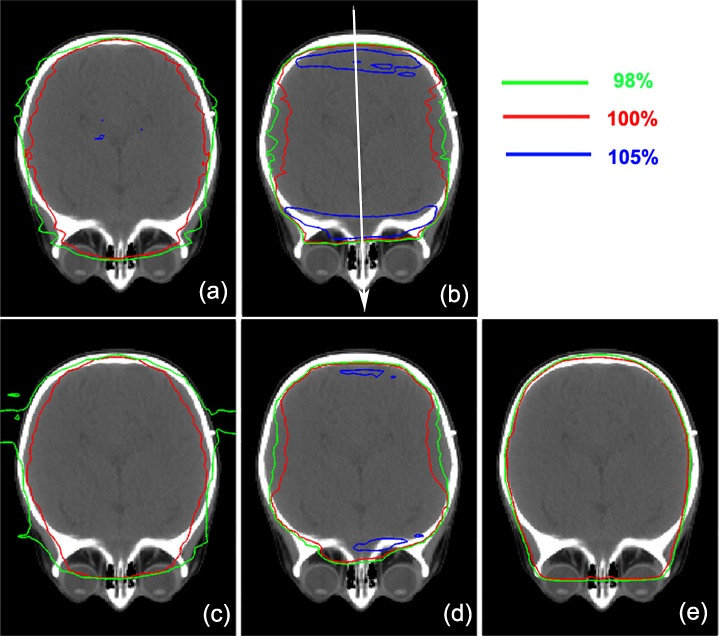
2D dose distributions near the subfrontal cribriform plate level in an axial view for patient #1 (listed in Table 1). Proton plans of the Eclipse and the XiO TPS with and without using compensator are shown: (a) Eclipse without compensator, (b) Eclipse with compensator, (c) XiO without compensator, and (d) XiO with compensator. A photon plan of the Pinnacle^3^ TPS is shown in panel (e). Isodose lines of 105%, 100% and 98% with respect to prescription dose $D_{P}$ (23.4 Gy (RBE)) are shown.

Calculated two‐dimensional (2D) dose distributions of the proton and photon plans near the subfrontal cribriform plate in an axial view are shown in Fig. 2 for patient #1 (listed in Table 1). For patient #1, with prescription dose $\left(D_{P}\right)$ of 23.4 Gy (RBE), no field modification was required to lower dose to lens for the proton plans with and without a compensator. By examining the isodose lines of 105%, 100% and 98% of $D_{P}$, the photon plan provided more uniform dose with a larger dose to eyes as compared to the proton plans. In the photon plan, nonuniform dose distributions resulting from varied attenuation through different thicknesses of tissue were compensated by using multiple segments.

The proton plans without a compensator gave similar high doses to eyes. However, isodose lines of 98% and 100% were further separated from a central axis of beam to anterior or posterior direction, and then the lines moved close to each other in anterior or posterior regions in both Eclipse and XiO ((Figs. 2a) and (c)). A larger separation between 98% and 100% isodose line was observed at the central axis. Due to lack of scattered protons caused by less tissue near anterior and posterior regions, sharper dose gradient was generated and forced the isodose lines of 98% and 100% to move closer.

The doses to eyes were further reduced in proton plans by using compensator with the result of larger dose nonuniformity to the brain and increased risk of potential underdosage to subfrontal areas due to setup errors, as shown in Fig. 2(b) and (d). Distinct hot spots (more than 105% of $D_{p}$) near anterior and posterior regions of brains were observed while reduced doses (within 95% of $D_{p}$) were seen at proximal and distal edges along the proton beam path. When a compensator was used, the distal edges of protons were determined by the distal edge of the PTV. To match the proton distal edge to the PTV automatically done by TPSs, the thickness of the compensator was increased from the central to the anterior/posterior region. As a result, a tighter margin between the PTV and 95% of the prescribed isodose line was generally observed in the subfrontal regions (less than 2 mm) in all of the patients, while a full aperture margin between them was shown in the compensator‐free proton and photon plans (about 8.0 mm). A thicker compensator generated larger fluence of scattered protons. In the UFPTI beam line (Eclipse modeling), the lateral displacement of protons in medium is predicted with the root mean square of the angular distributions of the proton trajectories (a fluence‐dose model)^(^ ^20^ ^,^ ^21^ ^)^ and in the MPRI beam line (XiO modeling), the $5{g/\text{cm}}^{2}$ water‐equivalent Lucite compensator increases the penumbra width (20%–80%) by about 2.5 mm.^(22)^ In addition, because the compensator was placed upstream of the patient, scattered protons generated by a thicker compensator outside the PTV (i.e., whole brain) also increased doses near anterior/posterior regions. With wider lateral penumbra induced by the scattered protons, distinct hot spots were generated near both anterior and posterior regions. However, because a treatment cross section became smaller at the central region near distal edge, outward scattered protons are not balanced by inward scattered protons due to less tissue at this depth. Therefore, reduced doses (within 95% of $D_{P}$) are seen at proximal and distal edges along the proton beam path after combing two obliquely opposed proton beams.

Areas of hot spots over 5% of prescription doses were larger for Eclipse plans than for the XiO plans. The differences between the Eclipse and XiO TPSs were caused by effects of different penumbrae of lateral profiles and distal fall‐offs of the proton beam lines (as shown in Fig. 1), variations in constructing compensators, and modeling of scattered proton passing through a compensator.

**Table 2:** $D_{5\%}$ of left and right lenses for proton and photon plans.

*Patient No.*	*Eclipse*				*XiO*					
	*Without Compensator*		*With Compensator*		*Without Compensator*		*With Compensator*		${\text{Pinnacle}}^{3}$	
	*Left*	*Right*	*Left*	*Right*	*Left*	*Right*	*Left*	*Right*	*Left*	*Right*
1	10.8	10.5	4.1	3.1	12.1	11.8	3.8	3.4	13.8	12.3
2	7.6	7.3	3.4	3.4	10.0	8.5	1.8	0.7	7.9	4.6
3	9.5	6.0	3.4	2.2	11.9	8.6	3.6	2.4	7.4	6.4
4	9.4	8.7	7.7	9.2	11.8	12.5	3.0	3.6	6.5	5.6
5	10.3	15.7	3.9	5.0	12.3	15.8	4.2	6.3	7.9	11.7
6	14.3	12.1	8.9	6.2	10.4	14.9	6.9	7.4	9.3	10.2
7	19.3	18.6	8.5	10.1	21.7	23.5	9.4	10.8	15.2	13.4
8	15.5	14.8	6.3	6.3	14.4	16.7	5.6	4.7	10.8	9.5
9	13.0	10.1	6.0	7.9	12.1	13.4	7.5	8.3	12.5	10.4
Mean (% to $D_{p}$)^a^	52.2	49.2	24.8	25.3	55.4	59.7	21.7	22.6	43.3	39.9
SD (% of mean $D_{5\%}$)^a^	30.2	36.0	38.1	46.7	27.1	32.9	48.1	60.2	30.5	33.3

a Mean values of $D_{5\%}$ in percentage of $D_{p}$ and SDs in percentage with respect to mean $D_{5\%}$. Because the mean value of $D_{5\%}$ is only about half of $D_{P}$, the SDs of $D_{5\%}$ are represented better by the percentage to their corresponding mean values of $D_{5\%}$.

**Figure 3:**
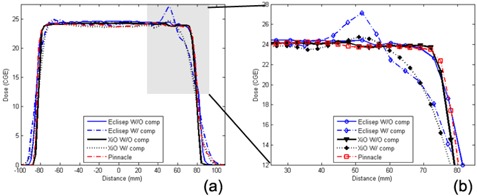
1D dose profiles of the proton and the photon plans passing through the cribriform plate. Detailed dose profiles at the anterior region are shown in panel (b) with expanded scales for both dose and distance. (abbreviations: W/=with, W/O=without, and comp=compensator)

To further examine the observed differences in 2D dose distributions between proton and photon plans, one‐dimensional (1D) dose profiles passing through cribriform plate along a white arrow (Fig. 2(b) were obtained (Fig. 3(a). Sharper 1D dose profiles in both anterior and posterior regions were observed for the proton plans without a compensator than those with a compensator. Sharp penumbra (5 mm) at the posterior region was caused by forcing doses to be zero at outside the patient's external contour and no correction of missing tissues, while the penumbra at the anterior region was 7 mm, as shown in Fig. 3(a).^(23)^

When a compensator was applied in a proton plan, width of full prescription dose was reduced by more than 10.0 mm, while width of 20% dose level was increased by 8.0 mm (Fig. 3(a). These results indicated that the penumbra width increases when a compensator was applied. The same width of full dose with similar width reduction in Eclipse and XiO indicated similar coverage of PTV in both proton plans.

We recognize that the width reduction was much larger at the anterior region than that at the posterior region (less than 3 mm). The increment of width for 20% dose level also occurred larger at the anterior region than that at the posterior region. Therefore, increased penumbra width was related not only to scattered protons generated by compensator but also to optimizing dose conformity to the complex shape of the PTV.

In the anterior region, the compensator was applied to encompass the cribriform plate (a narrow structure stuck out of the brain) with the distal edges of protons. Because the distal edges of protons were significantly pulled upstream for each proton beam in the plans with compensator in contrast to those without compensator, large decrement in the width of full dose was observed. In addition, because the cribriform plate is a narrow structure, small volumes receiving protons also extend to the 20% dose level away from the structures. In the posterior region, there is no narrow structure such as the cribriform plate. However, since the dose was forced to be zero at outside of patient's external contour, small variations in widths of full dose and 20% dose levels were seen. As a result, in both anterior and posterior regions, the penumbra width increased when the compensator is used. Both Eclipse and XiO showed similar tendencies, indicating that the penumbra width was not sensitive to the differences in forming compensator between them.

Hot spots were seen in the anterior and posterior regions (Fig. 2 (b) and (d)). The anterior regions generally showed a little higher dose than the posterior regions (Fig. 3(b) with expanded scale. Higher doses recorded in Eclipse than in XiO (as shown in 2D distributions) may have been caused by differences in modeling scattered protons generated by a compensator. Note that there is a potential risk of underdosage to the cribriform plate due to decreased width of full doses if a systematic position error occurs toward anterior. On the other hand, if the systematic position error is smaller than 2 mm, the cribriform plate can receive more than 105% of prescription dose. For the lens dose, even if there was a significant dose difference in lens with and without a compensator, the dose gradient along the anterior–posterior direction was similar in most of the cases (e.g., 115.1 cGy (RBE)/mm without a compensator and 119.7 cGy (RBE)/mm with a compensator for patient #7 in Table 1).

### B. DVHs and metrics of PTV and OARs

**Figure 4:**
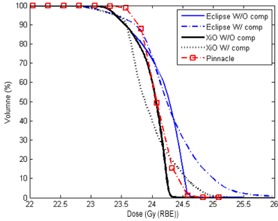
DVHs of the proton and the photon plans for the PTV of patient #1 (listed in Table 1). An expanded scale near the prescription dose is used in the dose axis.

Figure 4 shows extracted DVHs of PTV for the proton plans with and without a compensator in Eclipse and XiO and the photon plan in Pinnacle for patient #1 listed in Table 1. Because the doses to the isocenter was usually lower than doses to other locations away from isocenter for photon plan requiring $D_{P}$ doses to isocenter, 97% of PTV volume received the full $D_{P}$. Although large 15% nonuniformity was corrected, 10% of PTV volume still received more than 104% of $D_{P}$.

For the XiO proton plans with and without a compensator, the dose to isocenter was set to have 95% of PTV receiving $D_{P}$. In the XiO plan without a compensator, 50% of PTV volume received dose of 103% $D_{P}$ with a sharp DVH. Only 2% of PTV received more than 104% of $D_{P}$ compared to 20% of PTV for the photon plan. Applying a compensator to the XiO proton plan resulted in a long tail in DVH at doses over $D_{P};10\%$ of PTV volume received more than 105% of $D_{P}$.

Similar characteristics were seen in the Eclipse proton plan without a compensator, but a higher mean dose was required to have 95% of PTV receiving 100% of $D_{P}$, because of larger dose nonuniformity, which was attributed to the larger SSD effect for the proton line used in the Eclipse TPS. In the Eclipse proton plan without a compensator, 50% of PTV volume received 104% of $D_{P}$. The DVH for the proton plan with a compensator had a lower shoulder and a longer tail, and 10% of PTV volume received more than 107% of $D_{P}$. The longer tail in DVH means larger nonuniform dose delivered to PTV, observed in Fig. 2 as distinct hot spots.

Metrics (HI and $D_{2\%}$) of PTV for comparing the proton and photon plans are listed in Table 1. The mean values and standard deviations (SD) of HI of PTV for the proton plans without and with a compensator were 5.3%± 1.1% and 8.4%± 1.3% in the Eclipse TPS (p<0.0001), and 4.0%± 0.6% and 6.9%± 1.6% in the XiO TPS (p<0.0001), respectively, while the photon plans had 3.7%± 0.7%. Smaller value of HI means more uniform doses delivered to PTV, while smaller deviation of HI means less dependence on the patients’ anatomy. Only the proton plans without a compensator in XiO had mean values and SDs of HI comparable to those of the photon plans (p=0.34). Using compensators in the proton plans generated large mean values and SDs of HI, indicating large nonuniform doses delivered to PTV and strong dependence on the patients’ anatomy.

As shown in Table 1, there is dose gradient around PTV for both proton and photon plans, 2% of PTV $\left(D_{2\%}\right)$ usually receiving more than 5% of $D_{P}$. The mean values and SDs of $D_{2\%}$ of PTV for the proton plans without and with a compensator were 105.5%± 1.1% and 109.8%± 1.5% in the Eclipse TPS (p<0.0001), and 104.1%± 0.6% and 107.7%± 1.8% in the XiO TPS (p<0.0001), respectively, while the photon plans had 105.1%± 0.6%.

**Figure 5:**
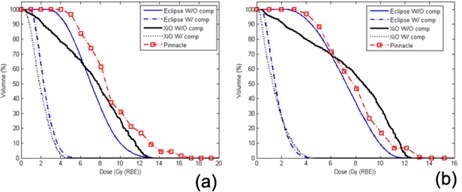
DVHs of the proton and the photon plans for left and right lenses of patient #1:(a) left and (b) right.

As shown in Fig. 5, DVHs of lens for the patient #1 were similar between the left and right lens for the Eclipse and XiO proton plans with a compensator. Doses to left and right lens were less than ~ 2.0 Gy (RBE) and ~ 0.5 Gy (RBE) to the 50% and 90% volumes of lens respectively, because the advantage of the distal edge of protons was utilized. For the proton plans without a compensator in both XiO and Eclipse, doses to the lenses were further increased to ~ 8.0 Gy (RBE) and ~ 4.0 Gy (RBE) to the 50% and 90% volumes of lens, respectively. It turned out that the DVHs of the proton plans without a compensator were very similar to that of the photon plan. However, dose to the 90% volumes of lens for the photon plan was further increased to 7.0 Gy in order to maintain the full prescription dose to the cribriform plate. Similar trends were observed in the other patients. Metrics $\left(D_{5\%}\right)$ of left and right lens to compare the proton and photon plans are listed in Table 2. The mean values (as a percentage of $D_{P}$) of $D_{5\%}$ to lens for the proton plans with and without a compensator were ~ 25% and ~ 51% in Eclipse and ~ 22% and ~ 57% in XiO, respectively, while the photon plan was ~ 42%. Because the mean value of $D_{5\%}$ was only about half of $D_{P}$, the SDs of $D_{5\%}$ were presented better by the percentage of their corresponding mean values of $D_{5\%}$. The SDs for the proton plans with and without a compensator were ~ 42% and ~ 33% in Eclipse and ~ 54% and ~ 30% in XiO, respectively, while the photon plan was ~ 32%.

Although larger volumes of normal tissue surrounding eyes received up to the full dose, $D_{5\%}$ was only around 40% of $D_{P}$ because the lenses were located more than 4 mm away from block edge where scattered photons and electrons were limited. However, large $D_{5\%}$ (up to more than half of $D_{P}$) were observed for the proton plans without a compensator, because one of two proton beams passed through all or part of the entire lens. Using a compensator on the proton plans kept protons from passing through the lens by pulling the distal edge away from the lens. To assure sufficient doses to the subfrontal cribriform plate and the brain tissues near eyes, $D_{5\%}$ could be only reduced by half with the finite width of the distal fall‐off. One should recognize that $D_{5\%}$ for the proton plans with a compensator is more sensitive to the uncertainties of proton beam ranges than the proton plans without a compensator and the photon plan. The maximum range prediction uncertainty of therapeutic proton energy without a compensator in the IBA beam line is less than 2.0 mm.^(24)^ Uncertainty due to conversion of HU to proton stopping power^(15)^ for a compensator is also included, when the compensator is in the beam line. Similar to SD of $D_{2\%}$ and HI of PTV, $D_{5\%}$ doses to lens are strongly dependent on individual patient's anatomy for all proton and photon plans. Large SD up to 50% of mean values of $D_{5\%}$ for the proton plans with a compensator indicates an extreme dependence of the treatment plans on individual patient's anatomy.

**Figure 6:**
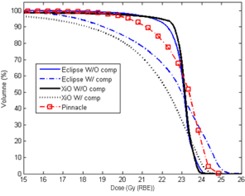
DVHs of the skull for the proton and the photon plans of patient #1.

Because isodose lines encompassed well the skull in the proton plans without a compensator than those with a compensator (Fig. 2 (b) and (d)), sharp DVHs of the skull near $D_{P}$ for the compensator‐free plans were seen (Fig. 6). A sharper dose gradient in the skull region and a wider shoulder of DVH were seen using the photon plan in comparison with the proton plans without a compensator. Although the compensator resulted in tight coverage of the whole brain (i.e., PTV) with the prescription dose, dose hot spots were generated near the skull at the anterior and posterior regions, and the 100% isodose line was dented inwardly at the central region along the beam path. The DVHs of the skull were slightly elongated in the proton plans with a compensator with lower shoulder for both Eclipse and XiO, as compared to those without a compensator. Because the hot spot was larger in the Eclipse plan with a compensator, 10% volume of the skull received 105% of $D_{P}$ for Eclipse versus 103% in Pianncle^3^ and 101% in XiO. The compensator forced the 100% isodose line to tightly encompass the whole brain inside the skull; the 90% volumes of the skull received only 85% and 75% of $D_{P}$ in the Eclipse and XiO plans, respectively. Table 3 compares proton and photon plans metrics $(D_{\text{mean}}$ and $D_{5\%}$) of the skull for selected patients. The mean values and SDs of mean doses of the skull were 97.8%± 0.4% and 98.9%± 1.2% for the proton plans without a compensator, while they were 95.6%± 1.5% and 93.9%± 2.2% for those with a compensator in Eclipse (p=0.0045) and XiO (p=0.031), respectively. The mean dose for all of the photon plans was 98.3%± 1.2%. The mean dose was reduced by more than 2% in the proton plans with a compensator, as compared to those without a compensator. The mean dose of the proton plans without a compensator was similar to the mean doses of photon plans (p>0.05 in all cases). The mean $D_{5\%}$ of the skull of the proton plans without a compensator was almost identical between Eclipse and XiO. The mean $D_{5\%}$ was increased by only 1% for the XiO proton plans with a compensator as compared to those without a compensator (p=0.034), but it increased by 5% for Eclipse (p<0.0001). The mean $D_{5\%}$ of the photon plans was 2% higher than the proton plans without a compensator. Although the difference is relatively small (only a few percent), it should be noted that the accuracy of the dose calculation on the surface and/or buildup regions of the TPSs is questionable where the equilibrium of scattered electrons or protons is not established.

**Table 3:** $D_{5\%}$ of the skull for proton and photon plans of selected patients.

*Patient No.*	*Eclipse*				*XiO*					
	*Without Compensator*		*With Compensator*		*Without Compensator*		*With Compensator*		${\text{Pinnacle}}^{3}$	
	$D_{\text{mean}}$	$D_{5\%}$	$D_{\text{mean}}$	$D_{5\%}$	$D_{\text{mean}}$	$D_{5\%}$	$D_{\text{mean}}$	$D_{5\%}$	$D_{\text{mean}}$	$D_{5\%}$
1	22.9	23.8	22.3	24.9	22.8	23.7	21.4	24.1	22.9	24.3
2	23.0	23.6	22.8	24.6	23.4	24.0	22.3	24.1	22.8	24.1
3	22.8	23.9	22.0	24.7	‐	‐	‐	‐	23.6	24.2
5	22.9	23.5	22.0	25.1	23.2	23.7	22.3	24.2	22.8	24.1
6	22.9	23.7	22.7	25.1	‐	‐	‐	‐	23.0	24.2
8	22.8	23.5	22.4	25.3	‐	‐	‐	‐	23.0	24.3
Mean (% to $D_{p}$)^a^	97.8	101.3	95.6	106.7	98.9	101.8	93.9	103.1	98.3	103.4
SD (% to $D_{p}$)^a^	0.4	0.7	1.5	1.1	1.2	0.8	2.2	0.4	1.2	0.4

a Mean values and SDs of D5% in percentage of Dp.

## IV. CONCLUSIONS

The dosimetric characteristics of WBRT by therapeutic proton plans was evaluated using the DVHs of PTV (i.e., whole brain), lens and skull and the 1D and 2D dose distributions of nine pediatric CSI patients. The prescribed isodose line encompassed the cribriform plate better in the proton plans with a compensator compared to either the proton plans without a compensator or the photon plans. Decreases of 10%–20% of $D_{P}$ to lens and about 5% of $D_{\text{mean}}$ to the skull were achieved by proton plans using a compensator compared to either the proton plans without a compensator or the photon plans. However, using a compensator in the proton plans comes with poorer dose homogeneity and higher $D_{2\%}$ to PTV. There was incremental increase of 5% in $D_{2\%}$ of PTV when the compensator was used in the proton plans. Additional differences observed in doses to PTV, lens and skull between Eclipse and XiO in this study were caused by different penumbra of lateral profile and distal fall‐off depth doses for the proton beam lines used in two treatment systems (UFPTI and MPRI).

Overall, other than the improvement of over 20% dose reduction to lens by using a compensator, only a little improvement in doses to cribriform plate and skull was observed in the proton plans and it was always accompanied higher doses to small volumes of whole brain and skull. Because the improvement of using a compensator is limited (a few percent) and the advantageous feature of distal fall‐off of proton is not available for WBRT, the proton plans with a compensator showed no distinguishable improvement in comparison with those without a compensator. In addition, using a compensator is more sensitive to the uncertainty of the proton distal edge; therefore, the proton plans without a compensator are used in our Institution. A study incorporating the uncertainty of the proton distal edge is needed to further investigate the differences between the proton plans with and without a compensator.
